# Biomass carbon storages and carbon sequestration potentials of the Grain for Green Program‐Covered Forests in China

**DOI:** 10.1002/ece3.4228

**Published:** 2018-07-03

**Authors:** Kaibo Wang, Dongfeng Hu, Juan Deng, Zhouping Shangguan, Lei Deng

**Affiliations:** ^1^ State Key Laboratory of Loess and Quaternary Geology Institute of Earth Environment Chinese Academy of Sciences Xi'an China; ^2^ State Key Laboratory of Soil Erosion and Dryland Farming on the Loess Plateau Institute of Soil and Water Conservation Chinese Academy of Sciences and Ministry of Water Resources Yangling China; ^3^ Environmental Protection Agency of Shaanxi Province Xi'an China

**Keywords:** carbon sequestration potential, carbon storage, China, Grain for Green

## Abstract

The Grain for Green Program (GGP) was the most all‐embracing program of ecological reconstruction implemented in China. To estimate carbon storages and carbon sequestration potentials of the GGP forests, the study presented in the paper collected data spanning from 1999 to 2010, such as tree species, tree planting area relevant to the GGP, empirical growth curves suitable for different planted tree species in China, as well as wood density (WD), biomass expansion factor (BEF), carbon fraction (CF) of different trees species, and estimated the carbon storages of the biomasses of GGP forests from 1999 to 2050. It showed that the total carbon storage of the biomass of GGP forests was 320.29 Tg upon the GGP completion in 2010; the total carbon sequestration is higher during the early GGP‐implementation stage than at the late GGP‐implementation stage, and the annual mean carbon sequestration of GGP forests was 26.69 Tg/year. The potential of GGP forests as carbon sink presented an increasing increment. In China, the potential increments of GGP forests as carbon sinks were estimated to be 397.34, 604.00, 725.53, and 808.90 Tg in 2020, 2030, 2040, and 2050, respectively, and the carbon sequestration rates were 1.72, 0.89, 0.52, and 0.36 Mg ha^−1^ year^−1^, respectively, corresponding to 2010s, 2020s, 2030s, and 2040s. Therefore, the GGP forests had bigger carbon sequestration capacities and potentials in China.

## 1 INTRODUCTION

Carbon dioxide (CO_2_) is one of the main greenhouse gases in the atmosphere (Zahn, 2009). It is predicted that by 2,100, atmospheric CO_2_ concentration will have nearly double relative to 100 years ago, causing the global temperature to increase by 1.0–3.7°C, sea level to rise, polar ice to melt, and lands to be disastrously submerged (IPCC, 2014). Forests as important carbon sinks of CO_2_ play an irreplaceable role in regulating global carbon balance, decreasing CO_2_ and other greenhouse gas in the atmosphere, and conserving the global climate (Deng, Han, Zhang, Tang, & Shangguan, 2017; He, Shifley, & Thompson, 2011; Houghton, 2007). In recent years, scientists all over the world have been discussing and estimating carbon sequestration capacities of global and regional forest ecosystems (Deng, Liu, et al., 2017; Härkönen, Lehtonen, Eerikainen, Peltoniemi, & Mäkelä, 2011; Heimann & Reichstein, 2008).

The international community pays high attention to carbon sequestration capacities forests in China. At present, there are many Chinese scientists exploring carbon storages of (Fang, Chen, Peng, Zhao, & Ci, 2001; Zhao & Zhou, 2006), carbon sequestration (Li & Lei, 2010), carbon sequestration potential of forest vegetation (Chen, Zhang, Zhang, & Wan, 2009), as well as some others focusing on carbon storages (Yu et al., 2007), carbon sequestration (Deng, Liu, & Shangguan, 2014), and carbon sequestration potential of forestland soils (Chang, Fu, Liu, & Liu, 2011; Zhang, Dang, Tan, Cheng, & Zhang, 2010) in China. However, there are a few researches on carbon sequestration capacities of plantations in China (Liu et al., 2014). As the biggest developing country of the world, China had the largest plantation area in the world (Peng et al., 2014). Since the 1980s, China government had carried out six major forestry programs, making its new plantation area gradually expand, so that these programs had played not only an important role in ecological environment improvement but also a positive role in atmospheric CO_2_ fixation (Deng, Liu, et al., 2017). However, currently, there are few studies conducted on effects of major forestry policies and forestry programs of China on it forest carbon sequestration capacities (Yin, Yin, & Li, 2010).

The Grain for Green Program (GGP) of China was one most all‐embracing ecological reconstruction program implemented by China (Deng & Shangguan, 2011), which afforested the largest area of forests (He et al., 2011). By its large‐scale forestations, the Program had established large areas of new forest vegetation, hence enhancing sequestration capacities of terrestrial ecosystems in China (Chen et al., 2009; Deng et al., 2014), and consequently, its effects on carbon sequestration potential of forests should not be ignored (Chen et al., 2009). At present, effects on the GGP forests as carbon sinks are mainly investigated in specific regions or in terms of forestland soil in China. Chen et al. (2009) studied effects of the GGP on forest carbon sequestration potentials in Yunnan of China. Chang et al. (2011) studied soil organic carbon sequestration potentials of the GGP‐covered Loess Plateau. And Zhang et al. (2010) researched on effects of the GGP on soil organic carbon sequestration rate in China. Although Liu et al. (2014) have estimated the changes in carbon fluxes and stocks caused by GGP using a process‐based ecosystem model, there are still limited researches carried out on effects of the GGP on carbon storages and carbon sequestration potentials of forest biomasses in China as a whole using inventory‐based method.

Therefore, in order to correctly assess effects of relevant forestry policies and forestry programs on forest carbon sequestration and in the hope of providing theoretical basis and data supports for plantation ecological assessment, the study investigated effects of the GGP on forest carbon sequestration capacities by estimating biomass carbon storages of GGP forest biomasses after the GGP implementation. The study was mainly intended to investigate effects of the GGP on (a) forest carbon storages, (b) carbon sequestration capacities, and (c) carbon sequestration potentials in the future 40 years.

## 2 MATERIALS AND METHODS

### 2.1 Program profile

**Figure 1:**
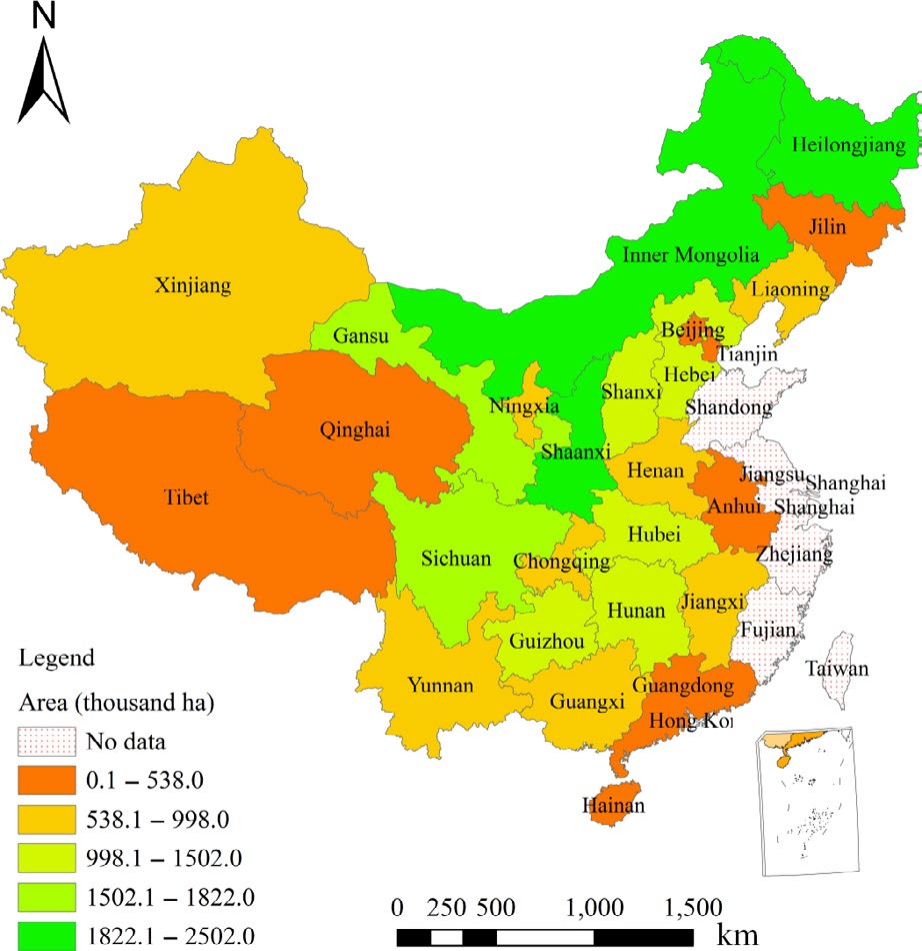
Coverage of the Grain for Green Program in China in 2010. Note: Data represent accumulative areas of croplands and barren land‐converted forestlands of GGP‐covered provinces from 1999 to 2010

In China, the Grain for Green Program was implemented for more than 10 years as a measure to control soil erosion (Deng & Shangguan, 2011). Launched in Shaanxi in 1999, the Program had become a nationwide program by 2000. Upon its completion in 2010, the Program had converted 14.67 million ha of farmlands into forestlands and grasslands and planted trees on 17.3 million ha of barren mountains and lands suitable for afforestation (Figure 1). As a result, it had transformed most steep slopelands into forestlands and brought the most severe desertificated farmlands under control. It had increased the grassland and forestland coverage of China by 4.5%. Therefore, it has greatly improved natural environments in China.

### 2.2 Geographical zones

**Figure 2:**
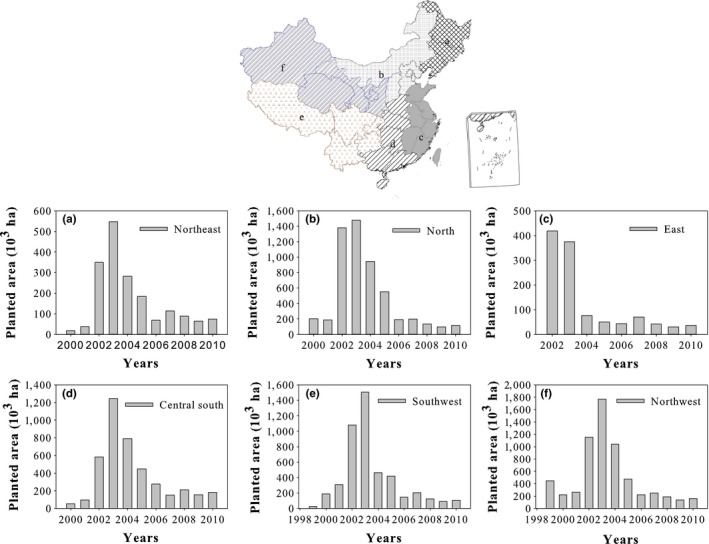
The six main Grain for Green Program‐covered regions of China from 1999 to 2010. Note: (a) Northeast China; (b) North China; (c) East China; (d) Central south China; (e) Southwest China; and (f) Northwest China

According to Fang's classification (Fang et al., 2001), China is divided into six geographical regions: Northeast China, North China, East China, Central south China, Southwest China, and Northwest China (Figure 2). Northeast China includes Heilongjiang, Jilin, Liaoning; North China includes Beijing, Hebei, Inner Mongolia, Shanxi, and Tianjin; East China includes Anhui, Fujian, Jiangshu, Jiangxi, Shandong, Shanghai, Taiwan, and Zhejiang; Central south China consists of Guangdong, Guangxi, Hainan, Henan, Hubei, Hunan, Hong Kong, and Macao; Southwest China is composed of Chongqing, Guizhou, Sichuan, Tibet, and Yunnan; and Northwest China included Gansu, Ningxia, Qinghai, Shaanxi, and Xinjiang. Figure 2 shows the coverage of the Grain for Green Program in the six geographical regions in China from 1999 to 2010.

### 2.3 Sources of the total GGP‐implemented areas and planting areas of individual tree species/forest types in China

1999–2010 annual data of the 31 provinces and municipalities of China, including those on land uses relevant to the Program and the Sandstorm Control Program around Beijing and Tianjin, were collected. 1999–2008 areas of farmlands and barren land‐converted forestlands of China by the GGP were cited from the China Forestry Statistical Yearbook (2000–2009), and 2009–2010 areas of farmlands and barren land‐converted forestlands of China by the GGP were cited from the China Forestry development bulletin (2010–2011). These data involved both the croplands and barren land‐converted forestlands of China.

The planting areas of the individual tree species/forest types of China were calculated by the GGP‐covered regions times the proportions of their forestation areas in the corresponding regions. The proportions of the forestation areas of the individual tree species/forest types of the regions were cited from GGP investigation reports, governmental forestry development reports, and relevant published literature on the different regions.

The GGP mostly afforested on barren hills and farmlands with slope gradient >15°, of which the soil was relatively poor and the soil moisture was lower, so that the forestation survival rates were unlikely to reach 100%. Thus, a correction factor, namely actual afforestation survival rate, was introduced. China's State Forestry Administration (SFA) survey (SFA, 2005) showed that the afforestation survival rate was only 90.2%, and thus, the actual planting areas were the results of the cited afforestation areas times the correction factor of 0.902.

### 2.4 Carbon storage estimation

Because the objects of the study were short‐time plantations, the carbon storages of their dead woods and litters were too low to take into account although they would increase after cropland or barren land afforestation or reforestation. Therefore, the study only took carbon storages of forest biomasses into account in its carbon storage estimation.

#### 2.4.1 Carbon storage estimation of forest biomasses

In general, carbon storages of forest vegetations are estimated by measuring and then multiplying their biomasses with their corresponding carbon fractions (CF). The carbon storage estimations of forest biomasses dynamics are related to the estimation of forest biomass changes. This study employed the empirical growth curve of plantations to estimate carbon storages of forest biomasses. The formulae for carbon storage estimation are as follows:

(1) $$C_{\mathrm{Bi}}=\sum_{j}\sum_{k}S_{\mathrm{jk}}V_{\mathrm{ijk}}D_{j}{\text{BEF}}_{j}{\mathrm{CF}}_{j}$$

(2) $$\text{or}\;C_{\mathrm{Bi}}=\sum_{j}\sum_{k}S_{\mathrm{jk}}B_{\mathrm{ijk}}{\text{CF}}_{j}$$

In which, *C*_*Bi*_ stands for carbon stocks (Mg) in living tree biomass in year *i*;* S*_*jk*_ stands for the area (ha) of species *j* planted or to be planted in year *k*;* V*_*ijk*_ stands for stand volume per hectare(m^3^/ha) of species *j* planted in year *k* in year *i*;* B*_*ijk*_ stands for stand biomass per hectare(Mg/ha) of species *j* planted in year *k* in year *i* for; *D*_*j*_ stands for the basic wood density (Mg/m^3^); BEF_*j*_ stand for the biomass expansion factor to convert stem biomass of species *j* into its stand biomass (whole trees, including stems, branches, foliages, and roots); and CF_*j*_ stands for the carbon fraction of species *j*. Equation (1) is suitable for arbor forests, and Equation (2) is suitable for shrub forests.

##### Stand volume or biomass estimation

In the model of carbon storage change in forest biomass estimation, stand volume (*V*) is a function change over time (forest age). The study adopted allometric growth equations of stand volume suitable for local Chinese plantation tree species/forest types published in the literature to estimate their stand volume. At present, there are not allometric growth equations for individual species developed so far, but there are only several groups of empirical curves capable of representing some main tree species/forest types. Those empirical curves all were the best ones screened depending on relevant published papers (Table 1). Dependent on the curves collected in the papers, the curves could be matched with only a few tree species/forest types (Table 1), and approximate alternatives can be matched with the other tree species/forest types (Chen et al., 2009). The empirical curves of coniferous species could be used as the curves of coniferous mixed forests; the empirical curves of hard broad‐leaved species could be used as the curves of broad‐leaved mixed forests; and the empirical curves of soft broad‐leaved species could be used as the curves of as the comprehensive curve of multiple tree species. The relation of shrub forest biomasses with forest age was obtained by referring to published papers. The study collected 96 groups of paired data of shrub biomasses at different forest ages and established the best nonlinear equation between forest biomass and forest age depended on them (Table 1). In addition, the study assumed that the age of a forest was one when it was afforested.

**Table 1:** Allometric growth equations of stand volumes or tree biomasses suitable for local Chinese plantation tree species/forest types

Tree species/Forest type	Growth curve	*R*	Sample size	*SE*	Reference
*Pinus massoniana* Lamb	*V* = 23.37 (1−e^−0.1023t^)^3.9135^	0.998	‐	‐	Tan et al., (1996)
*Cunninghamia lanceolata* (Lamb)Hook.	*V* = 308.64 (1−e−^0.1216t^)^4.2178^	0.962	306	‐	Zang, 2006
*Pinus elliottii* Engelm	*V* = 1,231.86 (1−e^−0.0041t^)^3.3939^	0.96	223	‐	Zhang, Chen, Luo, Gao, and Wu (2002)
*Pinus armandii* Franch	*V* = 139.94 (1−e^−0.0308t^)^1.9855^	‐	‐	2.529	Chen and Cai (2008)
*Pinus yunnanensis* Franch.	*V* = 161.42 (1−e^−0.0263t^)^1.6955^	‐	‐	5.424	Chen and Cai (2008)
Coniferous mixed forest	*V* = 178.06 (1−e^−0.0181t^)^0.9133^	‐	‐	2.071	Chen and Cai (2008)
Broad‐leaved mixed forest	*V* = 135.32 (1−e^−0.01434t^)^0.8859^	‐	‐	5.264	Chen and Cai (2008)
Multiple tree species comprehensive	*V* = 113.37 (1−e^−0.0486t^)^1.2526^	‐	‐	4.643	Chen and Cai (2008)
*Populus*	*V* = 365.50 (1−e^−0.1848t^)^3.9547^	0.999	‐	‐	Zhao, Zai, Hao, Tao, and Lu (2010)
*Eucalyptus* spp	*V* = 208.29 (1−e^−0.3320t^)^2.0767^	0.997	‐	‐	Zhou, Xie, and Liu (2005)
Shrub	*B* = 12.01/(1 + e^(2.5940−1.0823t)^)	0.618	96	4.282	This study

##### Biomass expansion factor, wood density, and carbon content

The study cited its basic wood densities (WD) and biomass expansion factors (BEF) from China initial national communication (Table 2) (IFEEP, 2004), and the carbon fractions (CF) were cited from the literatures (Table 2) (Li & Lei, 2010).

**Table 2:** Wood densities, biomass expansion factors, carbon fractions of different tree species/forest type

Tree species/Forest type	Wood density (Mg/m^3^)	Biomass expansion factor (by the whole tree)	Carbon fraction
*Pinus massoniana* Lamb	0.49	1.74	0.5221
*Pinus armandii* Franch	0.396	2.29	0.5225
*Pinus yunnanensis* Franch.	0.483	2.04	0.5113
*Pinus kesiya*	0.454	1.83	0.5224
*Abies fabric* (*Mast*).Craib	0.366	2.12	0.4999
*Picea asperata* Mast	0.342	2.12	0.5208
*Keteleeria fortunei* (Murr) Carr	0.448	2.23	0.4997
*Cryptomeria fortunei* Hooibrenk ex Otto et Dietr.	0.294	1.91	0.5201
*Cunninghamia lanceolata* (Lamb.)Hook.	0.307	1.92	0.5201
*Cupressus funebris* Endl.	0.478	2.11	0.5034
Coniferous mixed forest and other coniferous	0.405	2	0.5101
*Cinnamomum camphora* (L.) Presl.	0.46	1.89	0.4916
*Quercus*	0.676	2.09	0.5004
Hard broad‐leaved forest	0.598	2.34	0.4834
*Betula*	0.541	1.62	0.4914
*Sassafras tsumu*	0.477	2.49	0.4848
*Eucalyptus* spp	0.578	1.65	0.5223
*Populus*	0.378	2.16	0.4956
*Paulownia*	0.239	3.69	0.4695
Soft broad‐leaved forest	0.443	2.5	0.4956
Broad‐leaved mixed forest	0.482	1.95	0.49

#### 2.4.2 Carbon sequestration rate estimation

The method for carbon accumulation rate was adopted as follows (Zhou et al., 2008):

(3) $$\text{Cr}=\frac{Ctk-Ctj}{t_{k-j}},\left(k>j\right)$$

where Cr represents carbon accumulation rate (Mg ha^−1^ year^−1^); C_*tk*,_ and *C*_*tj*_ are carbon storage in year *k* and *j* (Mg); *t*_*k*‐*j*_ is the interval of time between years *k* and *j*. In our study, the Cr in the periods of 2010–2020, 2020–2030, 2030–2040, and 2040–2050 was the mean values of the 10 years of each period.

#### 2.4.3 Definition of carbon sequestration potential

Because of the GGP came to an end in 2010, the study estimated future potentials of GGP forests as carbon sinks assuming that the areas of the forests have been kept unchanged and the Chinese government had not fell the forests since 2010. The carbon sequestration potential of an ecosystem (potential carbon sink increment) is defined as the net carbon sequestration increment on the basis of benchmark carbon levels by an impact or management of natural factors or human factors. The study took 2010 as its baseline year for predicting the potentials of the GGP forests as carbon sinks in the following 40 years.

### 2.5 Data analysis

One‐way ANOVA was used to analyze the means of the each period among the different periods and different regions. Differences were evaluated at the 0.05 significance level. When significance was observed at the *p *<* *0.05 level, LSD (least significant difference) post hoc test was used to carry out the multiple comparisons. The data used for one‐way ANOVA have passed the homogeneity test. All statistical analyses were performed using the software program SPSS, ver. 17.0 (SPSS Inc., Chicago, IL, USA).

## 3 RESULTS

### 3.1 Carbon storage of forest biomass

The biomass carbon sequestration of China was 320.29 Tg (Table 3) upon the completion of the GGP in 2010, and higher at the late GGP‐implementation stage (2005–2010) than at the early GGP‐implementation stage (1999–2004), mainly because trees planted at the early GGP‐implementation stage grew bigger and bigger thus increasing the forest carbon storages of China. Because the GGP‐covered regions had different planting areas of GGP forests and tree species, they had different spatial distributions in carbon storages of forest biomass. The forest carbon sequestration of Central south China was the highest, reaching 80.26 Tg (Table 3), accounting for 25.06% of the total forest carbon sequestration of China, and the forest carbon sequestration of East China was the lowest, reaching 22.39 Tg (Table 3) and accounting for about 7% of the total forest carbon sequestration of China. The forest carbon sequestrations of the six regions varied consistently with those of China, that is, the forest carbon sequestrations were higher at the late GGP‐implementation stage (2005–2010) than at the early GGP‐implementation stage (1999–2004) (Table 3).

**Table 3:** Carbon storages of the biomasses of GGP forests from 1999 to 2010

Region	Year	Area (M ha)	Carbon storage (Tg)	Annual carbon sequestration (Tg/year)	Main tree species
Northeast	2000–2004	1.23	4.02	0.8	*Pinus koraiensis* Sieb. et Zucc., *Pinus sylvestris var*. mongolicaLitv, *Larix gmelinii* (Rupr.) Rupr. *Picea asperata* Mast*,* *Fraxinus mandshurica* Rupr., *Populus*
	2005–2010	0.59	19.33	3.22	
	2000–2010	1.82	23.35	2.12	
North	2000–2004	4.19	14.09	2.82	*Picea asperata* Mast*,* *Betula platyphylla* Suk *Larix gmelinii* (Rupr.) Rupr. *Hippophae rhamnoides* Linn. *Caragana Korshinskii* Kom.
	2005–2010	1.29	45.98	7.66	
	2000–2010	5.48	60.07	5.46	
East	2002–2004	0.87	3.12	1.04	*Pinus elliottii* Engelm, *Alnus cremastogyne* Burk., *Liquidambar formosana* Hance *Cinnamomum camphora* (L.) Presl. *Populus*
	2005–2010	0.27	19.27	3.21	
	2002–2010	1.14	22.39	2.49	
Central south	2000–2004	2.77	7.36	1.47	*Platycladus orientalis* (Linn.) Franco, *Illicium verum*, *Castanea mollissima*, *Camptotheca acuminate,* *Populus*,* Robinia pseudoacacia* L
	2005–2010	1.43	72.9	12.15	
	2000–2010	4.2	80.26	7.3	
Southwest	1999–2004	3.57	17.86	2.98	*Pinus armandii* Franch, *Cunninghamia lanceolata* (Lamb) Hook., *Cupressus funebris* Endl., *Eucalyptus* spp, *Populus*
	2005–2010	1.09	51.45	8.58	
	1999–2010	4.66	69.31	5.78	
Northwest	1999–2004	4.89	17.61	2.94	*Platycladus orientalis* (Linn.) Franco, *Robinia pseudoacacia* L, *Prunus armeniaca* *Hippophae rhamnoides* Linn., *Caragana Korshinskii* Kom.
	2005–2010	1.43	47.3	7.88	
	1999–2010	6.32	64.91	5.41	
China	1999–2004	17.53	64.06	10.68	
	2005–2010	6.1	256.23	42.71	
	1999–2010	23.63	320.29	26.69	

*Note*

The GGP started in Southwest China and Northwest China in 1999, in Northeast China, North China and Central south China in 2000, and in East China in 2002. The early GGP‐implementation stage was from 1999 to 2004, and the late GGP‐implementation stage was from 2005 to 2010.

### 3.2 Annual carbon sequestration of forest biomass

**Figure 3:**
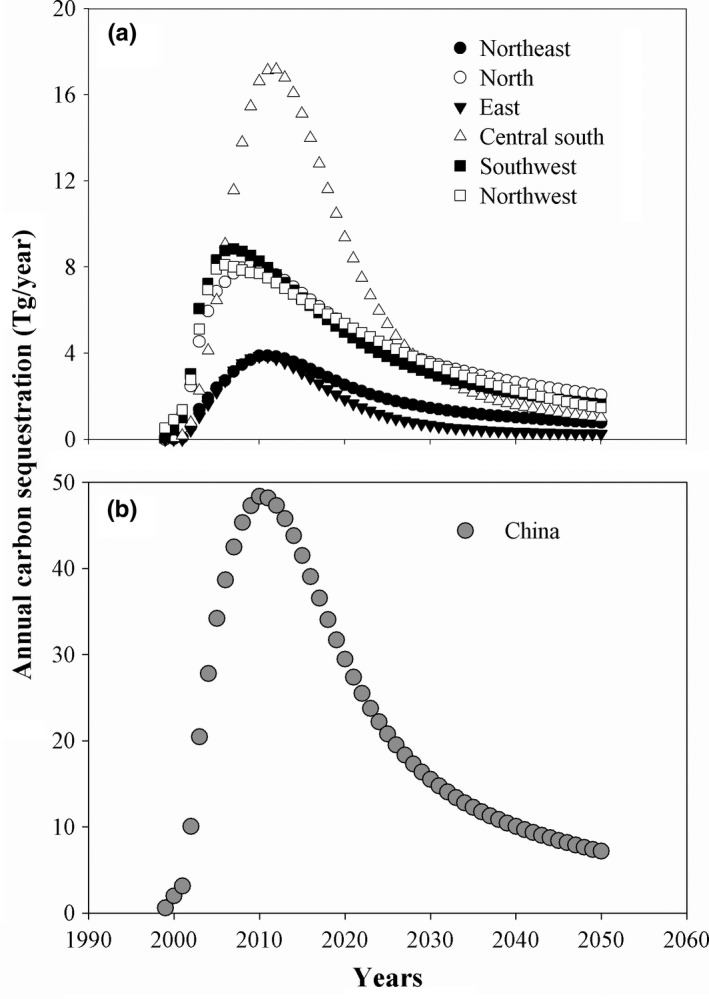
Annual carbon sequestrations of the biomasses of GGP forests in China and its six GGP regions

From 1999 to 2050, the annual carbon sequestrations of forest biomasses of China and the six GGP‐covered regions all showed a trend that the annual carbon sequestrations increased at the beginning, peaked around 2010 and decreased afterward, but the peaking times differed among the regions (Figure 3). From 2010 to 2050, the carbon sequestrations rates of forest biomasses of China and the six GGP‐covered regions showed in Table 4. The annual forest carbon sequestration of China reached its peak of 48.34 Tg in 2010. The annual mean carbon sequestration of forest biomasses of China was 26.69 Tg/year during the GGP‐implementation period from 1999 to 2010 and higher at the late GGP‐implementation stage (2005–2010) than at the early GGP‐implementation stage (1999–2004) (Table 3). Because the GGP‐covered regions had different planting areas of GGP forests and tree species, the GGP‐covered regions had different carbon storage properties in annual carbon storage of forest biomass. Like the carbon storage of forest biomass of the different GGP‐covered regions, the annual mean forest carbon sequestration of Central south China was the highest, reaching 7.30 Tg/year (Table 3), and the annual mean forest carbon sequestration of Northeast China was the lowest, reaching 2.12 Tg/year (Table 3) and accounting for about 7% of the total forest carbon sequestration of China. The forest carbon sequestrations of the six regions varied consistently with those of China, that is, the forest carbon sequestrations were higher at the late GGP‐implementation stage (2005–2010) than at the early GGP‐implementation stage (1999–2004) (Table 3).

**Table 4:** Carbon sequestrations rate (Mg ha^−1^ year^−1^) of the biomasses of GGP forests in China and its six GGP regions from 2010 to 2050

Year	Region						China
	Northeast	North	East	Central south	Southwest	Northwest	
2010–2020	1.80 ± 0.08aC	1.21 ± 0.05aE	2.57 ± 0.20aB	3.35 ± 0.22aA	1.38 ± 0.07aDE	1.09 ± 0.04aE	1.72 ± 0.09aCD
2020–2030	1.02 ± 0.05bB	0.78 ± 0.03bCD	0.94 ± 0.09bBC	1.28 ± 0.13bA	0.82 ± 0.04bBCD	0.73 ± 0.03bD	0.89 ± 0.05bBCD
2030–2040	0.65 ± 0.02cA	0.55 ± 0.02cB	0.41 ± 0.02cD	0.51 ± 0.03cBC	0.54 ± 0.02cB	0.47 ± 0.02cC	0.52 ± 0.02cBC
2040–2050	0.48 ± 0.01dA	0.42 ± 0.01 dB	0.26 ± 0.01cE	0.29 ± 0.01cDE	0.40 ± 0.01 dB	0.31 ± 0.01dD	0.36 ± 0.01dC

*Note*

The little letters indicate significant difference among different periods (*p* < 0.05); the capital letters indicate significant difference among different regions (*p* < 0.05).

### 3.3 Carbon sequestration potential of forest biomass

**Figure 4:**
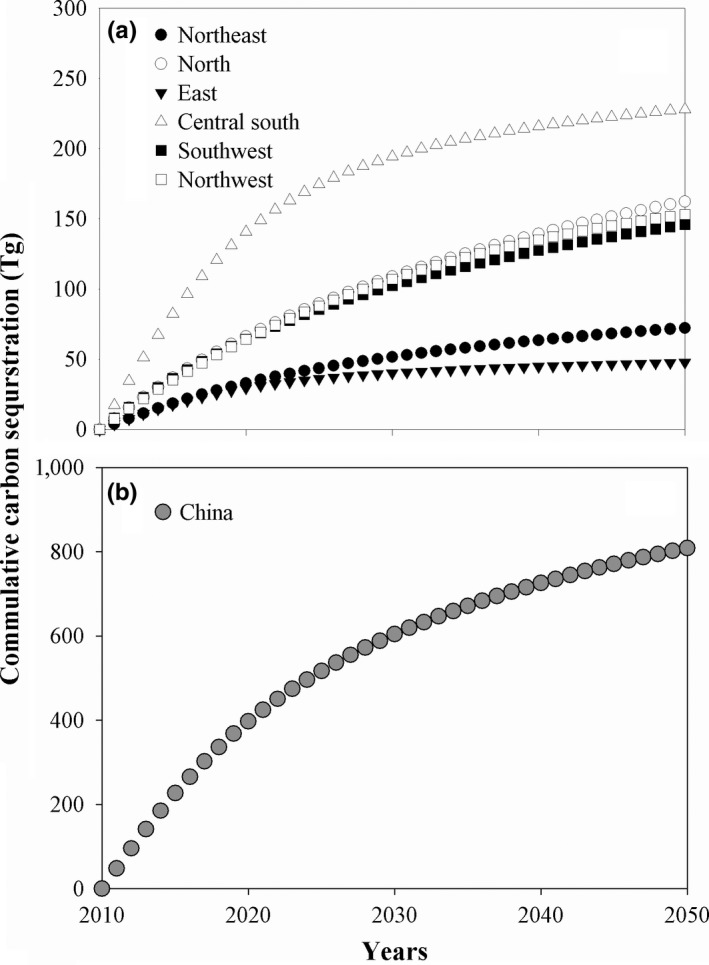
Carbon sequestration potentials of the biomasses of GGP forests in China and its six GGP regions

From the GGP completion in 2010–2050, the forest carbon sequestration potential of China will increase with time (Figure 4). The forest carbon sequestration potentials of China will be 397.34, 604.00, 725.53, and 808.90 Tg in 2020, 2030, 2040, and 2050, respectively. The forest carbon sequestration potentials of the six GGP‐covered regions all will increase with time, too. By 2050, the forest carbon sequestration potentials of Northeast China, North China, East China, Central south China, Southwest China, and Northwest China will have accounted for 8.92%, 20.06%, 5.90%, 28.18%, 18.02%, and 18.91% of the forest carbon sequestration potential of China, respectively.

## 4 DISCUSSION

### 4.1 The contribution of the GGP to carbon sinks in China

Forestry policies and forestry projects of China not only play an important role in improving its ecological environment but also modify its carbon sequestration capacity (Deng et al., 2014; Deng, Liu, et al., 2017; Wang et al., 2016). The carbon sequestration capacity due to implementation of forestry policies and forestry projects attract more and more attentions, and particularly after the Kyoto protocol took effect in 2005, which has prompted more countries and regions of the world carried out relevant researches (Deng et al., 2014; Jandl, Lindner, et al., 2007; Niu & Duiker, 2006). With its GGP implemented, the forest carbon storage estimates of China indicated that upon the GGP completion in 2010, the total carbon sequestration and annual mean carbon sequestration of China's GGP forests were 320.29 Tg and 26.69 Tg, respectively. The fixed carbon of China's GGP forests was equivalent to 14.56% of the total carbon emissions of China (calculated to be 2,200 Tg in 2010 [Durban conference]) during the same period of time. By 2050, the carbon sequestration potential of China's GGP forests will have reached 808.90 Tg, and thus, in 2050, the total carbon sequestration of the forests will be 1,129.19 Tg. The fixed carbon of China's GGP forests will be equivalent to 34.22%–47.05% of the total carbon emission of China (calculated to be 2,400–2,050 Tg in 2050 (Fang et al., 2009)) during the same period of time, or 47.45% of the total carbon emission of China that Ding, Duan, Ge, and Zhang (2009) estimated to be 2,380 Tg in 2050 provided that atmospheric CO_2_ concentration was kept below the target concentration of 470 ppm before the year. Therefore, the total carbon storage of China's GGP forests will be equivalent to 34.22%–47.45% of the total carbon emission in China during the same period of time. The results of the study showed that the GGP had higher carbon sequestration efficiency.

### 4.2 Tree growth equation accuracy

There are several methods in estimating forest biomass C stock at national or regional scales (Deng, Liu, et al., 2017), but no universally accepted approach for predicting future changes in forest biomass C stock (Xu, Guo, Piao, & Fang, 2010). The growth curves of forest volume (biomass) play a crucial role in estimating carbon storages in living tree biomasses (Chen et al., 2009; Deng, Liu, et al., 2017). Xu et al. (2010) have proved that the growth curves of forest volume method performed well in predicting the forest biomass C stocks dynamic at national scale. However, the estimation of future forest biomass C sequestration potential of GGP still has large difference (Deng, Liu, et al., 2017). In the current study, the potential increments of GGP forests as carbon sinks were estimated to be 397.34 and 808.90 Tg in 2020 and 2050, respectively. The results are higher than the estimation of Liu et al. (2014) but lower than the estimation of Deng, Liu, et al. (2017). The different results could mainly attribute to different methods used in estimating forest biomass C stock.

Moreover, the study adopted allometric growth equations of stand volumes (forest biomass) suitable for local Chinese plantation tree species/forest types to estimate their stand volume (forest biomass). At present, there are not allometric growth equations for individual species developed, but there are only several groups of empirical curves capable of representing some main tree species/forest types. Those empirical curves all are the best ones screened from the literatures (Table 1). Dependent on the curves collected in the papers, the empirical curves could be matched with only a few tree species/forest types (Table 1), and approximate alternatives can be matched with the other tree species/forest types. Due to restrictions of diverse climates, site conditions and management practices, etc., the carbon storages estimated by the growth curves would be higher under poor site conditions and lower in favorable site conditions (Chen et al., 2009). Thus, it is feasible to estimate carbon storage growths of GGP stands by designing prediction models of their forest carbons by these growth curves, considering that growth curves for different site conditions are unavailable. The carbon storage growths thus estimated are closer to their actual than those estimated by other methods. From the angle of improving model prediction precision, it will be necessary to further develop stand volume (forest biomass) growth models for various local tree species/forest types with time (forest age) in the future.

### 4.3 Carbon stock estimation of trees coarse wood residues

The study only estimated the biomass carbon storages of GGP forests. Due to lacks of relevant data, the study did not taken into account the effects of GGP on carbon storages of litters and dead woods. In general, farmland to forestland conversions will increase carbon storages of litters and dead woods to a certain extent. For a long period of time, the carbon storage of forest litters will increase obviously without forest logging, so that forest litters should not be ignored; and there will gradually appear dead standing trees, fallen logs, big diameter drying branches, and other coarse wood residues (Delaney, Brown, Lugo, Torres‐Lezama, & Quintero, 1998). Litter and dead woods will become important forest carbon pools. Thus, the two parts of GGP forests as carbon pools will significantly affect carbon sequestration potentials of the forests. Woods as raw materials are more and more fully exploited and recyclable economy is expanded, so that felled trees will be transformed as carbon pools in the forms of forest products rather than being immediately converted as carbon emissions. The forest products include building materials, decoration materials, furniture, and paper. Niu and Duiker (2006) reported that the carbon storage of wooden forest products could make up as high as 32% of the biomass carbon storage of felled trees from which the products were processed. So to cut GGP forests appropriately is helpful to extend the carbon sequestration capacities of the forests. In order to predict carbon potentials of GGP forests for a long time, further investigations and researches on litters and coarse wood residues as carbon pools as well as carbon storages of wooden forest products will be needed so as to estimate benefits of the GGP to increase forest products as carbon pools.

### 4.4 Forest managements

Because the study was a preliminary attempt to quantitatively assess ecological benefits of forestry policies and forestry projects of China and its main regions and in particular more and more attention was paid to the issue of carbon sequestration by man‐made management; therefore, the study still had many shortcomings. Because of lacks of relevant data and proper estimation methods, the study did not taken into account carbon sequestration benefits evolving from strengthening forest tending managements, pest control, fire prevention, etc. However, influence of management measures on carbon sequestration potentials should not be underestimated (Nabuurs et al., 2000). Considering hydrothermal condition influencing carbon balances of natural forests, plantations are a type of ecosystems under human control, and their operations and managements are more important factors that affecting their carbon balances (Jandl, Neumann, & Eckmullner, 2007; Jandl, Lindner, et al., 2007; Waterworth & Richards, 2008). Jandl, Neumann, et al. (2007) reported that forest managements could control carbon inputs and outputs by cutting, thinning, and intervening and optimized forest managements could not only maintain higher productivity but also increase forest soil carbon storage at the same time. Forest fires can increase soil carbon storage, but cause huge losses of biological carbon pools (Serrano‐Ortiz et al., 2011; van der Werf, Randerson, Collatz, & Giglio, 2003), and postdisaster managements play an important role in determining the function of forest ecological systems as carbon sinks (Serrano‐Ortiz et al., 2011). For example, postdisaster afforestation can quickly accelerate the transformation of ecosystems from carbon sources to carbon sinks (Merino, Real, Álvarez‐González, & Rodríguez‐Guitián, 2007). Incidences of forest diseases and insect pests as common biological natural disasters can severely harm forests. In 2010, China had 11.52 million ha of forests attacked by various hazards including forest diseases and insect pests, of which 87, 300 ha was most severely attacked (SFA, 2011). Incidences of forest diseases and insect pests indirectly affect forest soil carbon accumulation by directly influencing forest productivity. Incidences of forest diseases and insect pests can significantly reduce forest productivity, hence significantly reducing forest soil carbon accumulation. Therefore, strengthen managements of GGP forests, and promote implementations of their management measures, will greatly improve carbon sequestration capacities of the forests.

## 5 CONCLUSION

During the GGP implementation from 1999 to 2010, the total carbon sequestration was higher at the early stage than at the late stage. Upon the GGP completion, the forest carbon sequestration was 320.29 Tg, and the annual mean carbon sequestration was 26.69 Tg/year from 1999 to 2010. By 2050, the potential increment of GGP forests as carbon sinks will be 808.90 Tg. The study showed that GGP forests had higher carbon sequestration capacities and potentials. The annual mean carbon sequestration of GGP forest biomasses peaked in 2010. For afforestation, choosing tree species with higher carbon sequestration capacities and improving forest operations and managements can first produce bigger carbon benefits.

## AUTHORS’ CONTRIBUTIONS

Kaibo Wang, Zhouping Shanguan, and Lei Deng designed research and analyzed data; Dongfeng Hu and Juan Deng collected data and contributed to discussion; Kaibo Wang, Zhouping Shangguan, and Lei Deng wrote the paper.
